# Kinetic and Thermodynamic Characterization of Human 4-Oxo-l-proline Reductase Catalysis

**DOI:** 10.1021/acs.biochem.4c00721

**Published:** 2025-01-30

**Authors:** Ennio Pečaver, Greice M. Zickuhr, Teresa F. G. Machado, David J. Harrison, Rafael G. da Silva

**Affiliations:** † School of Biology, Biomedical Sciences Research Complex, University of St Andrews, St Andrews KY16 9ST, United Kingdom; § School of Medicine, University of St Andrews, St Andrews KY16 9TF, United Kingdom; ¥ EaStCHEM School of Chemistry, Biomedical Sciences Research Complex, University of St Andrews, St Andrews KY16 9ST, United Kingdom; £ NuCana Plc, Edinburgh EH12 9DT, United Kingdom

## Abstract

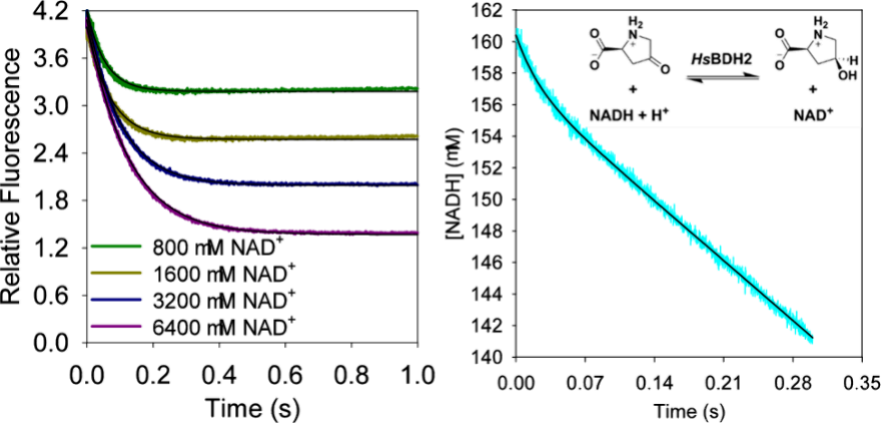

The enzyme 4-oxo-l-proline reductase (BDH2) has recently been identified in humans. BDH2, previously thought to be a cytosolic (*R*)-3-hydroxybutyrate dehydrogenase, actually catalyzes the NADH-dependent reduction of 4-oxo-l-proline to *cis*-4-hydroxy-l-proline, a compound with known anticancer activity. Here we provide an initial mechanistic characterization of the BDH2-catalyzed reaction. Haldane relationships show the reaction equilibrium strongly favors the formation of *cis*-4-hydroxy-l-proline. Stereospecific deuteration of NADH C4 coupled with mass spectrometry analysis of the reaction established that the pro*-S* hydrogen is transferred. NADH is co-purified with the enzyme, and a binding kinetics competition assays with NAD^+^ defined dissociation rate constants for NADH of 0.13 s^–1^ at 5 °C and 7.2 s^–1^ at 25 °C. Isothermal titration calorimetry at 25 °C defined equilibrium dissociation constants of 0.48 and 29 μM for the BDH2:NADH and BDH2:NAD^+^ complexes, respectively. Differential scanning fluorimetry showed BDH2 is highly thermostabilized by NADH and NAD^+^. The *k*_cat_/*K*_M_ pH–rate profile indicates that a group with a p*K*_a_ of 7.3 and possibly another with a p*K*_a_ of 8.7 must be deprotonated and protonated, respectively, for maximum binding of 4-oxo-l-proline and/or catalysis, while the *k*_cat_ profile is largely insensitive to pH in the pH range used. The single-turnover rate constant is only 2-fold higher than *k*_cat_. This agrees with a pre-steady-state burst of substrate consumption, suggesting that a step after chemistry, possibly product release, contributes to limit *k*_cat_. A modest solvent viscosity effect on *k*_cat_ indicates that this step is only partially diffusional. Taken together, these data suggest chemistry does not limit the reaction rate but may contribute to it.

## Introduction

Mammalian cytosolic protein BDH2, encoded by the *bdh2* gene, was initially thought to be an (*R*)-3-hydroxybutyrate dehydrogenase involved in ketone body metabolism, homologous to, yet distinct from, the *bdh1*-encoded mitochondrial (*R*)-3-hydroxybutyrate dehydrogenase known to be responsible for the interconversion between acetoacetate and (*R*)-3-hydroxybutyrate.^1^ In murine cells, BDH2 was demonstrated to be responsible for the synthesis of 2,5-dihydroxybenzoic acid, a mammalian siderophore, and *bdh2* expression downregulation causes iron imbalance in the cell.^2^ Recently, the human orthologue was also shown to accept l-2-keto-3-deoxyfuconate, a constituent of prokaryotic cell walls, as a substrate.^3^

BDH2 belongs to the short-chain dehydrogenase/reductase (SDR) superfamily, with a Rossmann fold for NAD(P)H binding and a characteristic Ser-Tyr-Lys catalytic triad.^1,4^ In SDRs, the Ser residue is proposed to position the substrate for catalysis while the Tyr residue acts as a general acid/base. The Tyr side-chain p*K*_a_ is lower than its typical solution value due to the proximity to the Lys residue.^5,6^ In human BDH2 (*Hs*BDH2), the Ser133-Tyr147-Lys151 triad is the catalytic triad, and the enzyme is specific for NAD^+^ over NADP^+^.^1^

**Scheme 1:**
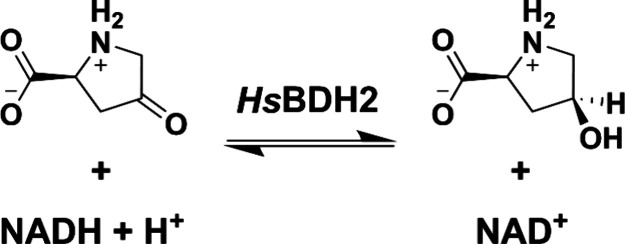
*Hs*BDH2-Catalyzed Reversible Reduction of 4-Oxo-l-proline

A seminal study showed *Hs*BDH2 to be a 4-oxo-l-proline reductase (EC 1.1.1.104), catalyzing the reversible, NADH-dependent reduction of 4-oxo-l-proline to *cis*-4-hydroxy-l-proline^7^ (Scheme 1), providing evidence for *cis*-4-hydroxy-l-proline synthesis in human cells.^7^ Interestingly, *cis*-4-hydroxy-l-proline is produced as the free amino acid^7^ as opposed to the post-translational modification of l-proline residues that generates *trans*-4-hydroxy-l-proline, catalyzed by prolyl hydroxylases.^8^ Importantly, *cis*-4-hydroxy-l-proline is a compound with known anticancer properties, having been the subject of preclinical and clinical studies against pancreatic cancer,^9−11^ even though its metabolic fate in normal cells and its specific mode of action against cancer are still elusive.^7,9−11^

Because of the importance of *Hs*BDH2 in the production of an endogenous anticancer metabolite, compounds that specifically modulate *Hs*BDH2 activity would be useful chemical tools to uncover the role of the enzyme in cancerous and healthy tissues. Understanding the biochemical features of the *Hs*BDH2-catalyzed synthesis of *cis*-4-hydroxy-l-proline could constitute an early step to enable inhibitor design. Here we employ UV–VIS absorbance and fluorescence spectroscopy, isothermal titration calorimetry (ITC), differential scanning fluorimetry (DSF), liquid chromatography-electrospray ionization-mass spectrometry (LC-ESI-MS), pH–rate profiles, steady-state kinetics, and pre-steady-state kinetics to investigate *Hs*BDH2-catalyzed 4-oxo-l-proline reduction. The interaction between *Hs*BDH2 and NADH is explored as well as the rate-limiting steps for the reaction.

## Materials and Methods

### Materials

All chemicals were used without further purification or modification. Ampicillin, dithiothreitol (DTT), and isopropyl β-d-1-thiogalactopyranoside (IPTG) were purchased from Formedium. *Escherichia coli* DH5α (high efficiency) and BL21(DE3) competent cells, Q5 DNA polymerase, and a Gibson Assembly Cloning Kit were purchased from New England Biolabs. Ethylenediaminetetraacetic acid-free complete protease inhibitor cocktail, 2-(*N*-morpholino)ethanesulfonic acid (MES), 4-oxo-l-proline, acetoacetate, lysozyme, 2-(cyclohexylamino)ethanesulfonic acid (CHES), and PEG-8000 were purchased from Merck. Agarose, 4-(2-hydroxyethyl)piperazine-1-ethanesulfonic acid (HEPES), *cis*-4-hydroxy-l-proline, dNTPs, and NaCl were purchased from ThermoFisher Scientific.

### Cloning and Expression of *Hs*BDH2

The DNA encoding *Hs*BDH2 (UniProt Q9BUT1) containing a tobacco etch virus protease-cleavable N-terminal six-His tag with restriction sites for NdeI and HindIII at the 5′- and 3′-ends, respectively, was purchased as gBlock with codons optimized for *E. coli* expression (IDT). The gBlock was inserted into a modified p*J*express414 vector plasmid via Gibson assembly^12^ following the manufacturer’s instructions (New England Biolabs). After Ni^2+^-affinity chromatography (described below), tobacco etch virus protease-catalyzed cleavage of the six-His tag was unsuccessful; thus, we decided to keep the six-His tag but remove the cleavage site from the final sequence. The construct was therefore modified by site-directed mutagenesis^13^ to remove the tobacco etch virus protease cleavage site to minimize deviation from the wild-type sequence. The forward primer 5′-TTCCATATGGGTCGTCTTGACGGGAAAGTAATTATCC-3′ and the reverse primer 5′-CGACCCATATGGAATTCCAAAAGTTAAACAAAATTATTTCTAG-3′ were used in the polymerase chain reaction (PCR) for site-directed mutagenesis, and the final protein sequence was MHHHHHHMGRLDGKVIILTAAAQGIGQAAALAFAREGAKVIATDINESKLQELEKYPGIQTRVLDVTKKKQIDQFANEVERLDVLFNVAGFVHHGTVLDCEEKDWDFSMNLNVRSMYLMIKAFLPKMLAQKSGNIINMSSVASSVKGVVNRCVYSTTKAAVIGLTKSVAADFIQQGIRCNCVCPGTVDTPSLQERIQARGNPEEARNDFLKRQKTGRFATAEEIAMLCVYLASDESAYVTGNPVIIDGGWSL.

The final construct was used to transform *E. coli* DH5α competent cells and sequenced (Eurofins) from plate colonies to verify mutation-free gene insertion. Then, the construct was used to transform BL21(DE3) competent cells (New England Biolabs). Transformed cells were grown in lysogeny broth supplemented with 100 μg mL^–1^ ampicillin at 37 °C until an optical density at 600 nm of 0.6–0.8 was reached. *Hs*BDH2 expression was induced with 0.5 mM IPTG, after which the cells were grown at 25 °C for an additional 16 h, harvested by centrifugation (6774*g* for 20 min at 4 °C), and stored at −20 °C.

### Purification of *Hs*BDH2

All chromatographic steps were performed using a AKTA Start FPLC system (Cytiva). Harvested cells were thawed on ice and resuspended in buffer A [50 mM HEPES, 500 mM NaCl, and 10 mM imidazole (pH 8.0)] supplemented with lysozyme (0.2 mg/mL), 0.05 mg/mL BaseMuncher (Expedeon), and a half-tablet of complete protease inhibitor cocktail (Roche). Resuspended cells were lysed in a cell disruptor (Constant Systems) at 30 000 psi and 5 °C and centrifuged (56000*g* for 20 min at 4 °C). The supernatant was filtered using a syringe with a 0.45 μm membrane filter and loaded onto a 5 mL HisTrap FF column (Cytiva) pre-equilibrated with buffer A.

For *Hs*BDH2 used in steady-state and pre-steady-state assays, chromatographic purification procedures were carried out at 4 °C. The column was washed with 15 column volumes (CV) of 8% buffer B [50 mM HEPES, 500 mM NaCl, and 500 mM imidazole (pH 8.0)]. Adsorbed protein was eluted with a 20 CV gradient of 8% to 70% buffer B. Fractions were collected and analyzed by sodium dodecyl sulfate–polyacrylamide gel electrophoresis (SDS–PAGE) in NuPAGE Bis-Tris 4–12% precast gels (ThermoFisher Scientific) alongside the PageRuler Plus Stained Protein Ladder (ThermoFisher Scientific). Fractions containing purified *Hs*BDH2 were collected, dialyzed against 2 × 2 L of buffer C [100 mM HEPES, 12.5 mM NaCl, and 1 mM DTT (pH 7.0)], and concentrated using a 10 000 molecular weight cutoff (MWCO) ultrafiltration membrane.

For *Hs*BDH2 stocks used in binding assays, the chromatographic steps were carried out at room temperature. The column was washed with 30 CV of 8% buffer B, and adsorbed protein was eluted with a 20 CV gradient from 8% to 70% buffer B. Fractions were collected and analyzed by SDS–PAGE as described above. Fractions containing purified *Hs*BDH2 were collected and dialyzed against 2 L of buffer C. The dialyzed protein solution was treated with 20 mM acetoacetate for 30 min at room temperature, washed with buffer, and concentrated in the ultrafiltration membrane. This process was repeated once, after which the protein solution was again dialyzed against 2 L of buffer C.

The concentrations of *Hs*BDH2 and NADH within the samples were determined spectrophotometrically in a Shimadzu UV-2600 spectrophotometer. The NADH concentration was determined at 340 nm (ε_340_ = 6220 M^–1^ cm^–1^). The *Hs*BDH2 concentration was determined at 280 nm (ε_280_ = 18 825 M^–1^ cm^–1^; ProteinParam tool of ExPasy) while controlling for NADH absorbance at the same wavelength (ε_280_ = 3600 M^–1^ cm^–1^).^14^*Hs*BDH2 was aliquoted and stored at −80 °C. The molecular mass of *Hs*BDH2 was verified via LC-ESI-MS analysis at the University of St Andrews Proteomics and Mass Spectrometry Facility.

### Synthesis of 4*S*-[4-^2^H]NADH and 4*R*-[4-^2^H]NADH

The biocatalytic synthesis and purification of 4*S*-[4-^2^H]NADH were performed as previously reported.^6^ Biocatalytic synthesis of 4*R*-[4-^2^H]NADH was achieved as previously described.^15,16^ Briefly, a solution containing 0.05 M sodium bicarbonate, 0.05 M deuterated formic acid (Merck), 0.015 M NAD^+^, and 1 mg of formate dehydrogenase at pH 8.5 was allowed to react at room temperature. After 4 h, the solution pH was adjusted to 8.5 again. Purification was carried out as previously described for 4*S*-[4-^2^H]NADH.^6^

### *Hs*BDH2 Activity Assay for Steady-State Kinetics

All 500 μL assays were monitored spectrophotometrically at 340 nm for 1 min under initial rate conditions in 1 cm optical path length quartz cuvettes (Hellma) using a Shimadzu UV-2600 spectrophotometer outfitted with a CPS unit for temperature control. In the forward direction, activity was monitored via the decrease in absorbance corresponding to NADH oxidation (ε_340_ = 6220 M^–1^ cm^–1^). In the reverse direction, activity was monitored via the increase in absorbance, indicating NAD^+^ reduction. Unless otherwise stated, experiments were carried out at 25 °C and pH 7.0. Reactions were carried out in assay buffer containing 100 mM HEPES (pH 7.0), 12.5 mM NaCl, and 1 mM DTT. In the forward direction, 6–20 nM *Hs*BDH2 was used, as well as varying concentrations of one substrate at a fixed, saturating concentration of the other (either 0–1024 μM 4-oxo-l-proline and 24 μM NADH or 3–24 μM NADH and 1024 μM 4-oxo-l-proline). For the reverse reaction, 20–40 nM *Hs*BDH2 was used, as well as varying concentrations of one substrate at a fixed, saturating concentration of the other (0–64 mM *cis*-4-hydroxy-l-proline and 3200 μM NAD^+^ or 0–3200 μM NAD^+^ and 64 mM *cis*-4-hydroxy-l-proline). The pH of the *cis*-4-hydroxy-l-proline stock solution was adjusted to 7.0. An additional experiment was carried out under previously published assay conditions.^7^ The reaction was performed at 37 °C in 80 mM sodium phosphate (pH 6.5), 1 mM DTT, and 0.1 mg mL^–1^ bovine serum albumin, with 4.4 nM *Hs*BDH2, 40 μM NADH, and 0–2048 μM 4-oxo-l-proline. Two independent rate measurements were carried out. Controls lacked the enzyme.

### LC-ESI-MS Analysis of the *Hs*BDH2-Catalyzed Reaction

A mixture of 300 μM NADH or its isotopologues, 300 μM 4-oxo-l-proline, and 10 μM *Hs*BDH2 in assay buffer was incubated at room temperature for 1 h, after which the enzyme was removed by centrifugation in 10 000 MWCO Vivaspin microfuge tubes. LC-ESI-MS analyses of the *Hs*BDH2-catalyzed reaction and reagent standards NADH, 4*S*-[4-^2^H]NADH and 4*R*-[4-^2^H]NADH were carried out on a Waters Atlantis Premier BEH C_18_ AX 1.7 μm, 2.1 mm × 100 mm column in an Acquity H-Class UPLC system coupled to a Waters Xevo G2-XS Q-TOF mass spectrometer. The flow rate was set to 300 μL min^–1^ with initial conditions of 90% 10 mM ammonium acetate (pH 6.0) and 10% acetonitrile for 0.5 min followed by a step change to 50% 10 mM ammonium acetate (pH 6.0), 40% 10 mM ammonium acetate (pH 10.0), and 10% acetonitrile for 2.0 min, and re-equilibration in 90% 10 mM ammonium acetate (pH 6.0) and 10% acetonitrile for 4.5 min. LC-ESI-MS analyses of the *Hs*BDH2-catalyzed reaction and reagent standards 4-oxo-l-proline and *cis*-4-hydroxy-l-proline were carried out on an ACE Excel 2 AQ, 2.1 mm × 100 mm column. The flow rate was set to 300 μL min^–1^ with initial conditions of 99% water/formic acid (0.1%) and 1% acetonitrile/formic acid (0.1%) for 1 min, followed by a linear gradient to 1% water/formic acid (0.1%) and 99% acetonitrile/formic acid (0.1%) for 1 min, and held until 3 min, followed by re-equilibration to 99% water/formic acid (0.1%) and 1% acetonitrile/formic acid (0.1%) for 5 min. The column oven temperature was maintained at 40 °C, and the injection volume was 5 μL. The ESI interface was operated in negative mode with a capillary voltage of 2.5 kV, a source temperature of 100 °C, a desolvation temperature of 250 °C, and a desolvation gas flow of 600 L h^–1^. Mass spectra were acquired in TOF mode in the range of *m*/*z* 90–800 at a rate of one scan per second.

### *Hs*BDH2 pH–Rate Profiles

The pH dependence of *k*_cat_ and *k*_cat_/*K*_M_ was determined by measuring initial rates in a composite buffer (100 mM MES, 100 mM HEPES, 100 mM CHES, 12.5 mM NaCl, and 1 mM DTT) in the pH range of 6.0–9.0, with 20 nM *Hs*BDH2 and 24 μM NADH when varying the concentration of 4-oxo-l-proline (from 0 to a maximum of 7100 μM, depending on the pH), and 6 nM *Hs*BDH2 and a saturating concentration of 4-oxo-l-proline when varying the concentration of NADH (6–24 μM). To ensure that *Hs*BDH2 remained stable at the pH extremes, enzyme stocks were diluted in buffer at pH 6.0 and 9.0 prior to measuring activity at pH 7.0, without any noticeable change in activity. Two independent rate measurements were carried out.

### *Hs*BDH2 Kinetics in the Presence of Glycerol

The effect of glycerol on *k*_cat_ and *k*_cat_/*K*_M_ was determined by measuring initial rates in the presence of 0–18% (v/v) glycerol. Initial rates were also measured in the presence of 5% (w/v) PEG-8000 as a control. Reaction mixtures contained 20 nM *Hs*BDH2 and 24 μM NADH when varying the concentration of 4-oxo-l-proline (0–2024 μM), and 6 nM *Hs*BDH2 and 2024 μM 4-oxo-l-proline when varying the concentration of NADH (6–24 μM). Two independent measurements were performed.

### Rapid Kinetics

All rapid kinetics experiments were performed in an Applied Photophysics SX-20 stopped-flow spectrophotometer outfitted with a 5 μL mixing cell (0.5 cm path length, 0.9 ms dead time) and a circulating water bath for temperature control. Reactions were triggered by rapid mixing of 55 μL of each syringe. For *Hs*BDH2 kinetics under single- and multiple-turnover conditions, reactions measured depletion of NADH at 25 °C via an absorbance decrease at 340 nm. Each syringe contained assay buffer. For each reaction, a minimum of four traces were collected and averaged. For single-turnover reactions, one syringe contained 20 μM *Hs*BDH2 and 20 μM NADH and the other contained either 2048 or 4096 μM 4-oxo-l-proline. Traces contained 9000 data points over 0.6 s in a logarithmic time scale. For the multiple-turnover experiments, one syringe contained 320 μM NADH and 16 μM *Hs*BDH2 and the other contained 4096 μM 4-oxo-l-proline. Traces contained 2400 data points over 0.3 s on a logarithmic time scale.

The NADH dissociation rate was measured via the decrease in fluorescence upon the departure of NADH with an excitation wavelength of 340 nm and an emission cutoff filter of 400 nm. Each syringe contained assay buffer. One syringe contained 20 μM *Hs*BDH2 and 16.6 μM NADH, and the other contained 1.6–12.8 mM NAD^+^. At 25 °C, fluorescence decay was monitored with 5000 data points collected over 1 s on a logarithmic time scale. At 5 °C, 5000–9000 data points were collected over 10 s in a logarithmic time scale. For each reaction, a minimum of five traces were collected and averaged.

### Thermostability of *Hs*BDH2

DSF-based thermal denaturation measurements were performed in 96-well plates on an Applied Biosystems QuantStudio 1 Real-Time PCR instrument. Reaction mixtures (20 μL) contained assay buffer and 5× SYPRO Orange (Invitrogen) with addition of either 0–160 μM NADH to 8 μM *Hs*BDH2 or 0–1200 μM NAD^+^ to 7.7 μM *Hs*BDH2 apoenzyme. Thermal denaturation was followed via concomitant fluorescence emission from SYPRO Orange (λ_ex_ = 520 ± 10 nm, and λ_em_ = 558 ± 11 nm) across a temperature range of 25–95 °C in increments of 0.05 °C s^–1^. Controls were performed in the absence of the enzyme and subtracted from measurements containing the enzyme. For each concentration of NADH or NAD^+^, at least two independent measurements were carried out. The thermostability of *Hs*BDH2 activity was determined in an Applied Photophysics SX-20 stopped-flow spectrophotometer. One syringe contained 600 nM *Hs*BDH2, and the other 48 μM NADH and 4096 μM 4-oxo-l-proline. The enzyme was continuously incubated within the syringe at 25 °C for 3.5 h, and rate data were collected every 0.5 h. Traces had 2000 data points collected over 10 s on a linear time scale.

### Isothermal titration calorimetry (ITC)

ITC measurements were carried out in a MicroCal PEAQ-ITC calorimeter (Malvern Instruments). Experiments were performed at 25 °C. NADH and NAD^+^ were solubilized in *Hs*BDH2 assay buffer at concentrations of 200 μM and 3 mM, respectively. After an initial injection of 0.4 μL, 19 successive 2 μL injections of the respective ligand were performed in a solution of 30 μM *Hs*BDH2 apoenzyme. Injections were carried out in 120 s intervals with a reference power of 10 μcal s^–1^. The heat of dilution was measured using a negative control, where the ligand was titrated into assay buffer, and these data were subtracted from the binding curve. Two independent experiments were performed for each ligand. Data were fitted to a single-site binding model (1:1 stoichiometry with no cooperativity) in the PEAQ-ITC analysis software (Malvern instruments). For NAD^+^ binding analysis, the number of sites was fixed at one.

### Kinetics and DSF Data Analysis

DSF and kinetics data were analyzed by the nonlinear regression function of SigmaPlot 13.0 (SPSS). Substrate saturation curves at a fixed concentration of the co-substrate were fitted to eq 1. The pH–rate profiles were fitted to eq 2. Protein denaturation data were fitted to eqs 3 and 4. Data for the dissociation rate constant by competition were fitted to eqs 5 and 6. The apparent *K*_eq_ was calculated with eq 7. Solvent viscosity effects were obtained from eq 8. Pre-steady-state kinetics under multiple- and single-turnover conditions were analyzed with eqs 9 and 10, respectively. In eqs 1–10, *v* is the initial rate, *k*_cat_ is the apparent steady-state catalytic rate constant, *K*_M_ is the apparent Michaelis constant, *E*_T_ is the total enzyme concentration, *S* is the concentration of the varied substrate when that of the co-substrate is held constant, *C* is the pH-independent value of *k*_cat_, *H* is the proton concentration, *K*_a1_ and *K*_a2_ are apparent acid dissociation constants, *F*_U_ is the fraction unfolded, *T* is the temperature in degrees Celsius, *T*_m_ is the melting temperature, *c* is the slope of the transition region, LL and UL are the folded and unfolded baselines, respectively, Δ*T*_m_^max^ is the maximum thermal stabilization, *L* is the ligand concentration, *K*_*T*_m__ is the apparent dissociation constant for L, *F*(*t*) and *F*_∞_ are fluorescence at times *t* and ∞, respectively, *A*_0_ is the signal amplitude, *k*_obs_ is the observed rate constant, *K*_eq_^app^ is the apparent equilibrium constant, *k*_cat_^0^ and *k*_cat_^η^ represent the *k*_cat_ in the absence and presence of glycerol, respectively, η_rel_ is the relative viscosity of the solution, *m* is the slope, NADH(*t*) and NADH_∞_ are NADH concentrations at times *t* and ∞, respectively, *k*_burst_ is the burst-phase rate constant, and *k*_STO_ is the single-turnover rate constant.
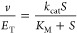
1
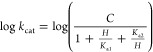
2
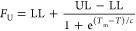
3
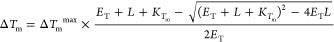
4
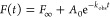
5
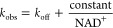
6
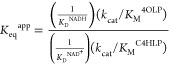
7
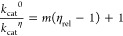
8
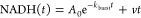
9
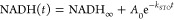
10

## Results

### Purification and Steady-State Kinetics of *Hs*BDH2

**Figure 1:**
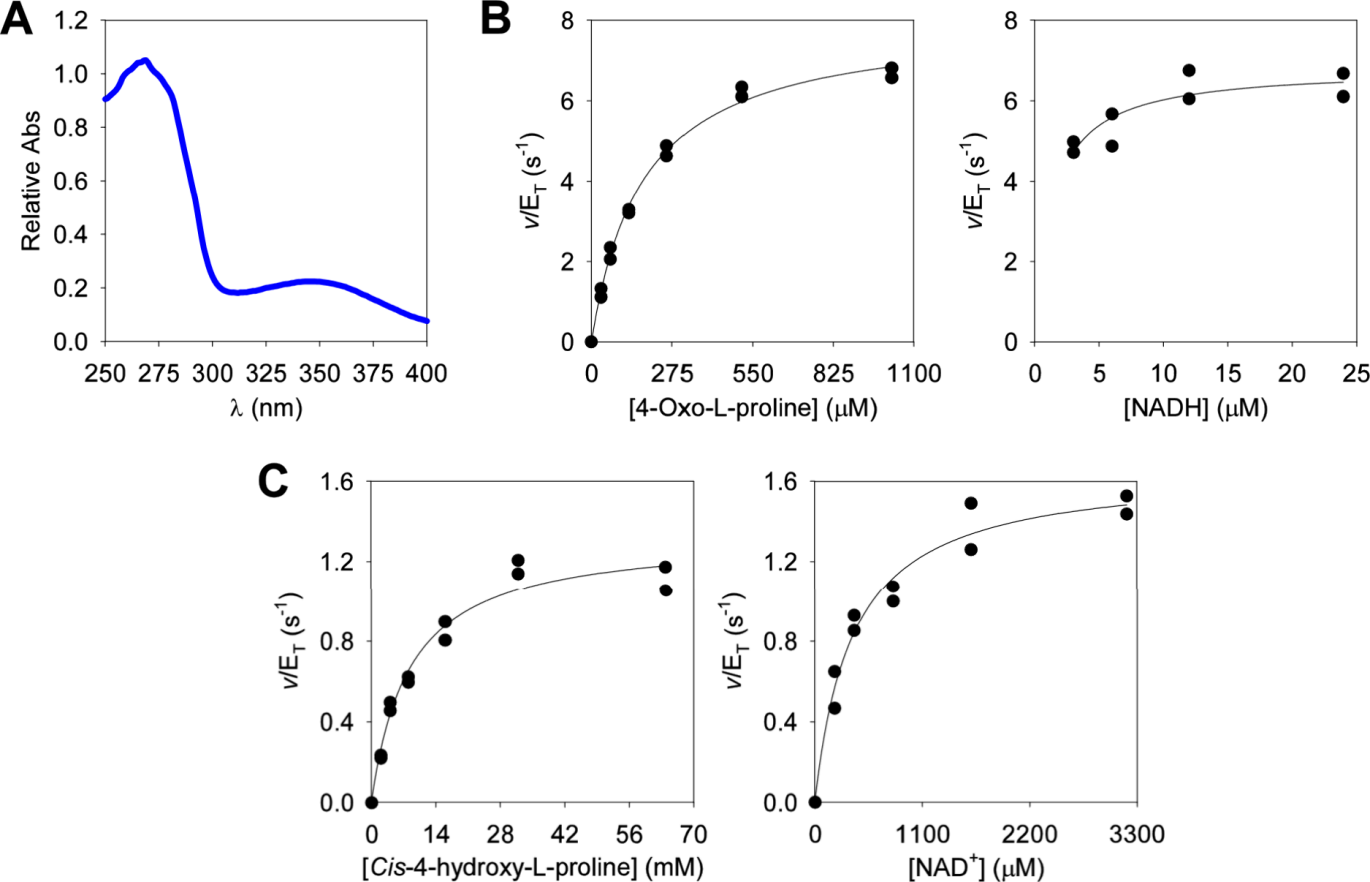
Biochemical characterization of *Hs*BDH2. (A) UV–VIS spectrum of purified *Hs*BDH2. (B) Michaelis–Menten plots for *Hs*BDH2-catalyzed reduction of 4-oxo-l-proline. (C) Michaelis–Menten plots for *Hs*BDH2-catalyzed oxidation of *cis*-4-hydroxy-l-proline. All Michaelis–Menten plots were determined at 25 °C and pH 7.0. All data points are shown for two independent measurements. Lines are the best fits of the data to eq 1.

*Hs*BDH2 was purified to homogeneity as judged by Coomassie Blue-stained SDS–PAGE (Figure S1). LC-ESI-MS analysis indicated that the mass of the purified protein matches the predicted mass of N-terminally His-tagged *Hs*BDH2 (Figure S2). A 4-oxo-l-proline saturation curve under experimental conditions reported previously (pH 6.5 and 37 °C)^7^ yielded apparent kinetic parameters (Figure S3) in excellent agreement with published values.^7^ The UV–VIS spectrum of purified *Hs*BDH2 revealed, in addition to the expected peak at 280 nm, a peak at 340 nm (Figure 1A), suggesting the purified enzyme was bound to NADH. Quantification of NADH and *Hs*BDH2 by their respective extinction coefficients estimated that ∼83% of the purified enzyme was bound to NADH, which was reproduced across several independent batches of *Hs*BDH2.

At 25 °C and pH 7.0, 4-oxo-l-proline and NADH saturation curves (Figure 1B) were fitted to eq 1 to yield an apparent *k*_cat_ of 7.4 ± 0.2 s^–1^ (mean ± propagated fitting error), an apparent *k*_cat_/*K*_M_^4OLP^ of (4.2 ± 0.3) × 10^4^ M^–1^ s^–1^ (value ± propagated fitting error), and an apparent *K*_M_^4OLP^ of 176 ± 12 μM (value ± fitting error). The *K*_M_^NADH^ was too low (*K*_M_^NADH^ < 3 μM) to be reliably estimated by fitting of the data to eq 1. However, fitting the data to eq 1 places a lower limit on the apparent *k*_cat_/*K*_M_^NADH^ of ∼2.5 × 10^6^ M^–1^ s^–1^. Saturation curves for *cis*-4-hydroxy-l-proline and NAD^+^ (Figure 1C) were fitted to eq 1 to generate an apparent *k*_cat_ of 1.50 ± 0.05 s^–1^ (mean ± propagated fitting error), an apparent *K*_M_^C4HLP^ of 8 ± 1 mM (value ± fitting error), an apparent *k*_cat_/*K*_M_^C4HLP^ of 188 ± 24 M^–1^ s^–1^ (value ± propagated fitting error), an apparent *K*_M_^NAD^+^^ of 394 ± 65 μM (value ± fitting error), and an apparent *k*_cat_/*K*_M_^NAD^+^^ of (3.8 ± 0.6) × 10^3^ M^–1^ s^–1^ (value ± fitting error).

### Acid–Base Chemistry in the *Hs*BDH2-Catalyzed Reaction

**Figure 2:**
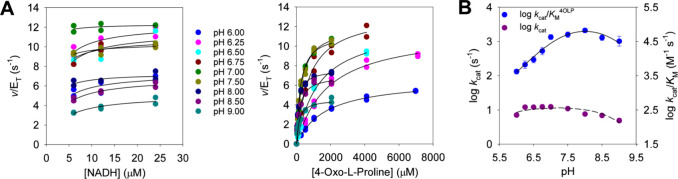
*Hs*BDH2 pH–rate profiles. (A) Michaelis–Menten plots were obtained for *Hs*BDH2 at different pH values. All Michaelis–Menten plots were determined at 25 °C. All data points are shown for two independent measurements. Lines are best fits of the data to eq 1. (B) *Hs*BDH2 pH–*k*_cat_/*K*_M_^4OLP^ and pH–*k*_cat_ profiles. Data points are means ± propagated fitting errors and values ± propagated fitting errors for the *k*_cat_ and *k*_cat_/*K*_M_^4OLP^ profiles, respectively. Lines are best fits of the data to eq 2.

To probe the role of acid–base chemistry in the NADH-dependent reduction of 4-oxo-l-proline, apparent steady-state parameters were determined at various pH values (Figure 2A), and pH–rate profiles were obtained for *k*_cat_ and *k*_cat_/*K*_M_^4OLP^ (Figure 2B). Because of the very low *K*_M_^NADH^, no profile could be accurately obtained for *k*_cat_/*K*_M_^NADH^. The *k*_cat_/*K*_M_^4OLP^ profile was bell-shaped; the best fit of the data to eq 2 indicated one group with an apparent p*K*_a_ of 7.3 ± 0.2 (value ± fitting error) must be deprotonated for the optimum binding of 4-oxo-l-proline to the *Hs*BDH2:NADH complex and/or for catalysis, while another group with an apparent p*K*_a_ of 8.7 ± 0.2 (value ± fitting error) must be protonated. The latter value must be considered with caution because there are not enough data points to define precisely the slope on the basic limb, which eq 2 assumes to be −1. The *k*_cat_ profile was less informative, and although the data were fitted to eq 2, neither acidic nor basic limb is well-defined in the pH range employed. This suggests that the relevant p*K*_a_ values are displaced outwardly in comparison with the *k*_cat_/*K*_M_^4OLP^ profile.

### Coenzyme Binding Confers Thermostability to *Hs*BDH2

**Figure 3:**
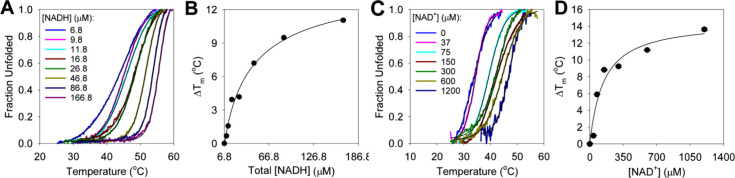
DSF-based thermal denaturation of *Hs*BDH2. (A) Denaturation of *Hs*BDH2 in the presence of NADH. (B) Dependence of Δ*T*_m_ on the NADH concentration. (C) Denaturation of the *Hs*BDH2 apoenzyme in the presence and absence of NAD^+^. (D) Dependence of Δ*T*_m_ on the NAD^+^ concentration. In panels A and C, each trace is the average of three measurements, and black lines are best fits of the data to eq 3. In panels B and D, data points are the values ± fitting errors, and lines are the best fits of the data to eq 4. All data were collected at pH 7.0.

To test whether coenzyme binding may induce thermostability to *Hs*BDH2, DSF-based thermal melting profiles were evaluated in the presence of increasing concentrations of either NADH (including 6.8 μM prebound to the protein) or NAD^+^ (Figure 3). Each thermal denaturation trace was fitted to eq 3 (Figure 3A,B) to produce a melting temperature (*T*_m_). The change in *T*_m_ as compared to the *T*_m_ without adding the coenzyme was plotted as a function of the concentration of either NADH (Figure 3B) or NAD^+^ (Figure 3D). At 166.8 μM NADH, for *Hs*BDH2 (∼83% of which are already bound to NADH at the beginning of the experiment) the *T*_m_ of 44.5 ± 0.1 °C increases to 55.5 ± 0.1 °C, giving a Δ*T*_m_ of 11 °C. The best fit of the data to eq 4 predicted a maximum Δ*T*_m_ of ∼20 °C (Figure 3B), probably an overestimation. The *Hs*BDH2 apoenzyme has a low *T*_m_ of 33.3 ± 0.1 °C (Figure 3C), and addition of NAD^+^ increases the *T*_m_ by a predicted maximum of ∼15 °C from the best fit of the data to eq 4 (Figure 3D).

### Dissociation Rate Constant of the *Hs*BDH2:NADH Complex

**Figure 4:**
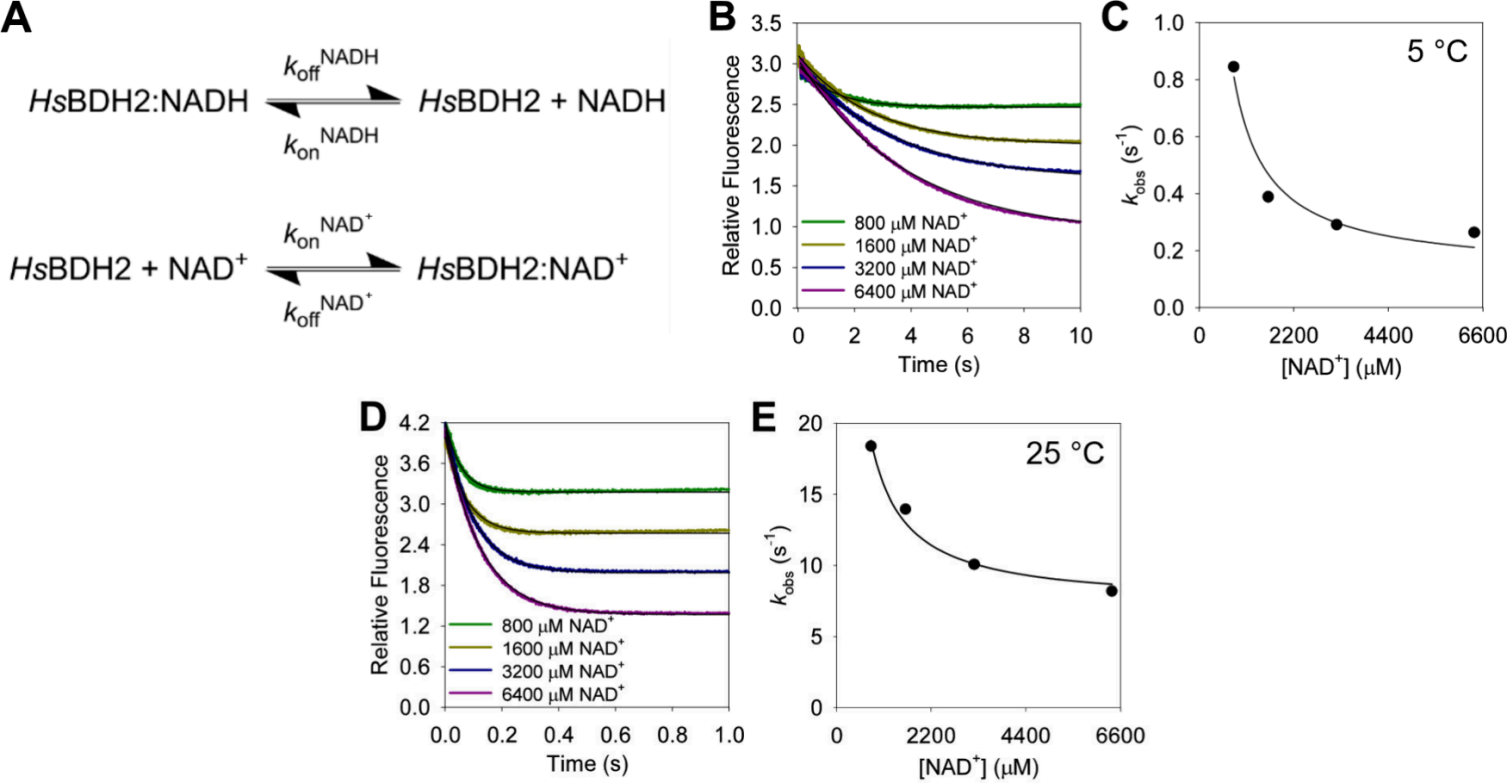
NADH dissociation rate by competition binding kinetics. (A) Reactions required to form the *Hs*BDH2:NAD^+^ complex from the *Hs*BDH2:NADH complex. (B) Time course of fluorescence decay upon rapid mixing of NAD^+^ and the *Hs*BDH2:NADH complex at 5 °C. (C) Dependence of the observed fluorescence decay rate constant at 5 °C on NAD^+^ concentration. (D) Time course of fluorescence decay upon rapid mixing of NAD^+^ and the *Hs*BDH2:NADH complex at 25 °C. (E) Dependence of the observed fluorescence decay rate constant at 25 °C on NAD^+^ concentration. In panels B and D, each trace is the average of 10 reactions, and thin black lines are best fits of the data to eq 5. In panels C and E, data points are values ± fitting errors, and the lines are best fits to eq 6. All data were collected at pH 7.0.

The co-purification of *Hs*BDH2 and NADH suggests the *Hs*BDH2:NADH binary complex dissociates slowly. To estimate the apparent dissociation rate constant (*k*_off_) for NADH, the rapid kinetics competition binding of NAD^+^ to *Hs*BDH2 was evaluated by following the fluorescence (λ_ex_ = 340 nm; λ_em_ > 400 nm) change observed upon the release of NADH from the enzyme (Figure 4). Considering the sequential reactions depicted in Figure 4A, *k*_off_^NADH^ will be rate-limiting for the formation of the *Hs*BDH2:NAD^+^ complex provided *k*_on_^NAD^+^^[NAD^+^] ≫ *k*_off_^NADH^ and *k*_on_^NAD^+^^[NAD^+^] ≫ *k*_on_^NADH^[NADH], a condition that can be met by increasing the NAD^+^ concentration until the observed rate constant for fluorescence decay becomes independent of the concentration of the competitor.^17^ Rapid mixing of NAD^+^ with the *Hs*BDH2:NADH complex at 5 °C led to exponential fluorescence decay (Figure 4B). The best fit of the data to eq 5 yielded the observed rate constants whose hyperbolic dependence on NAD^+^ concentration (Figure 4C) could be fitted to eq 6(17) to generate a *k*_off_^NADH^ of 0.13 ± 0.06 s^–1^. This low off rate likely underpins the purification of *Hs*BDH2 mostly bound to NADH, because the protocol is carried out at 4 °C. At 25 °C (Figure 4D,E), the *k*_off_^NADH^ increases ∼55-fold to 7.2 ± 0.7 s^–1^.

### *Hs*BDH2 Apoenzyme

To purify *Hs*BDH2 without bound NADH, three observations were important. The enzyme can catalyze the reduction of acetoacetate to generate (*R*)-3-hydroxybutyrate and NAD^+^, albeit with a very low *k*_cat_/*K*_M_.^1,7^ The *k*_off_^NADH^ is significantly increased at 25 °C in comparison with the value at 5 °C (Figure 4). Finally, the *Hs*BDH2 catalytic activity is stable with respect to incubation of the enzyme at 25 °C for ≥3.5 h (Figure S4). Using a protocol that included the chromatographic step carried out at room temperature (∼21 °C) and two rounds of reaction of the enzyme with excess acetoacetate at 25 °C followed by ultrafiltration at 4 °C, *Hs*BDH2 was isolated (Figure S5) and the expected mass was confirmed by LC-ESI-MS (Figure S6). UV–VIS spectroscopy indicated significant, but not complete, loss of NADH (Figure S7). Quantification of *Hs*BDH2 and NADH by their respective extinction coefficients estimated that ∼22% of the purified enzyme was bound to NADH, which was reproducible across subsequent preparations. This enzyme, mostly free of NADH, is termed the *Hs*BDH2 apoenzyme. The apparent steady-state kinetic parameters of the *Hs*BDH2 apoenzyme are in accordance with those of NADH-bound *Hs*BDH2 (Figure S8).

### Thermodynamics of the Coenzyme Binding to *Hs*BDH2

**Figure 5:**
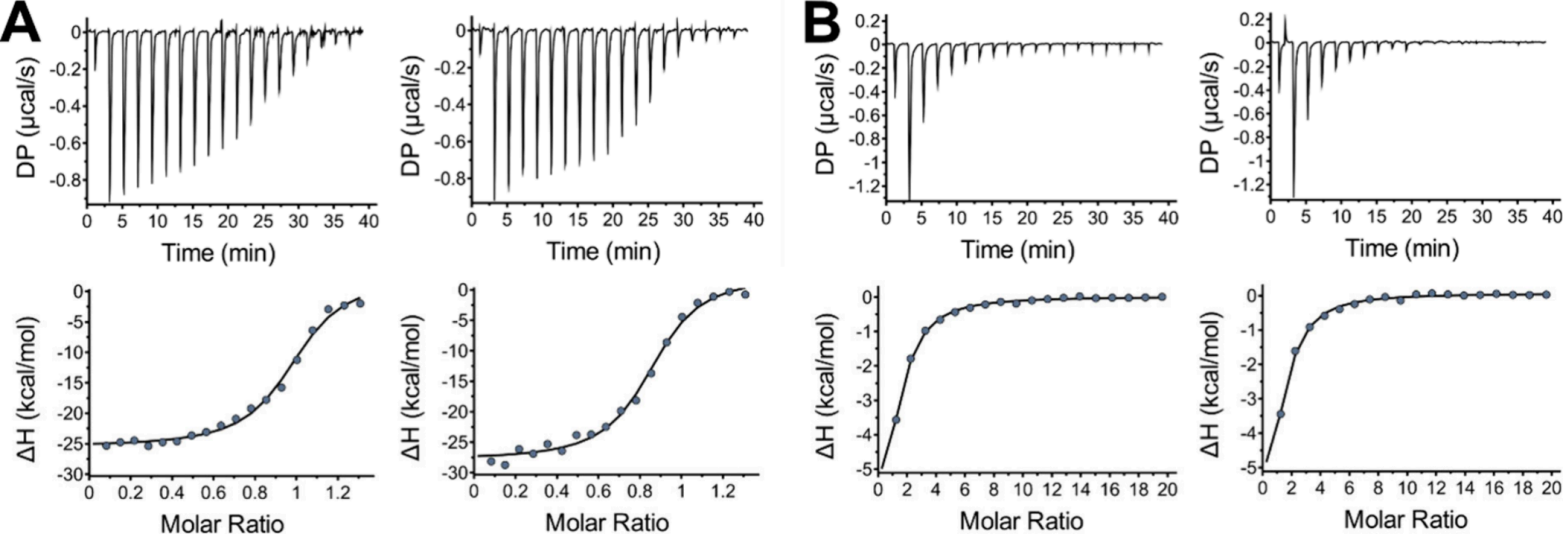
Coenzyme binding thermodynamics by ITC. (A) Two independent experiments of NADH titrated into an *Hs*BDH2 apoenzyme solution. (B) Two independent experiments of NAD^+^ titrated into an *Hs*BDH2 apoenzyme solution. All data were collected at 25 °C and pH 7.0 and best fitted to a single-site binding model with a 1:1 (protein:ligand) stoichiometry. The reported thermodynamic parameters are the mean of both experiments with each coenzyme.

The equilibrium binding of the *Hs*BDH2 apoenzyme to either NADH or NAD^+^ was investigated by ITC, using the concentration of NADH-free *Hs*BDH2 as the concentration of the enzyme available for binding. Both NADH and NAD^+^ binding isotherms were best fitted to a single-site model with a 1:1 (protein:ligand) stoichiometry (Figure 5). The equilibrium dissociation constant for NADH (*K*_D_^NADH^) is 0.48 ± 0.1 μM, whereas the *K*_D_^NAD^+^^ is 29 ± 2 μM. Gibbs free energies (Δ*G*) of −8.6 and −6.2 kcal/mol were obtained for NADH and NAD^+^ binding, respectively. The formation of each binary complex is exothermic and enthalpically driven, with a Δ*H* of −29 ± 1 kcal/mol and a *T*Δ*S* of −20.1 kcal/mol for NADH binding and a Δ*H* of −10.2 ± 0.3 kcal/mol and a *T*Δ*S* of −4.0 kcal/mol for NAD^+^ binding.

### Haldane Relationship and Reaction Equilibrium Constant

Assuming a sequential bi-bi kinetic mechanism for *Hs*BDH2, as is the case for several related (*R*)-3-hydroxybutyrate dehydrogenases,^6,18^ the apparent equilibrium constant (*K*_eq_) for the reversible reduction can be calculated from the *K*_D_ and apparent *k*_cat_/*K*_M_ values using the kinetic Haldane given by eq 7.^19^ An apparent *K*_eq_ of 13 497 ± 3800 at pH 7.0 and 25 °C was calculated, producing a true *K*_eq_ of ∼(1.4 ± 0.4) × 10^11^ M^–1^, because the true *K*_eq_ for this type of reaction is given by the apparent *K*_eq_/[H^+^].^20,21^ This points to the formation of *cis*-4-hydroxy-l-proline as being highly favorable at equilibrium with a Δ*G* of −15.2 kcal/mol.

### Transfer of the Pro*-S* Hydrogen from NADH

To determine whether *Hs*BDH2 catalyzes the transfer of the pro-*S* or pro-*R* hydrogen as a hydride from NADH to 4-oxo-l-proline, stereospecific deuteration of NADH and high-resolution LC-ESI-MS analysis were employed. Both 4*S*-[4-^2^H]NADH and 4*R*-[4-^2^H]NADH were enzymatically synthesized, and their masses were confirmed by high-resolution LC-ESI-MS, along with the masses of commercial NADH, 4-oxo-l-proline, and *cis*-4-hydroxy-l-proline (Figure S9A,B). No *cis*-4-hydroxy-l-proline was detected when NADH and 4-oxo-l-proline were allowed to react in the absence of *Hs*BDH2 (Figure S9C).

**Figure 6:**
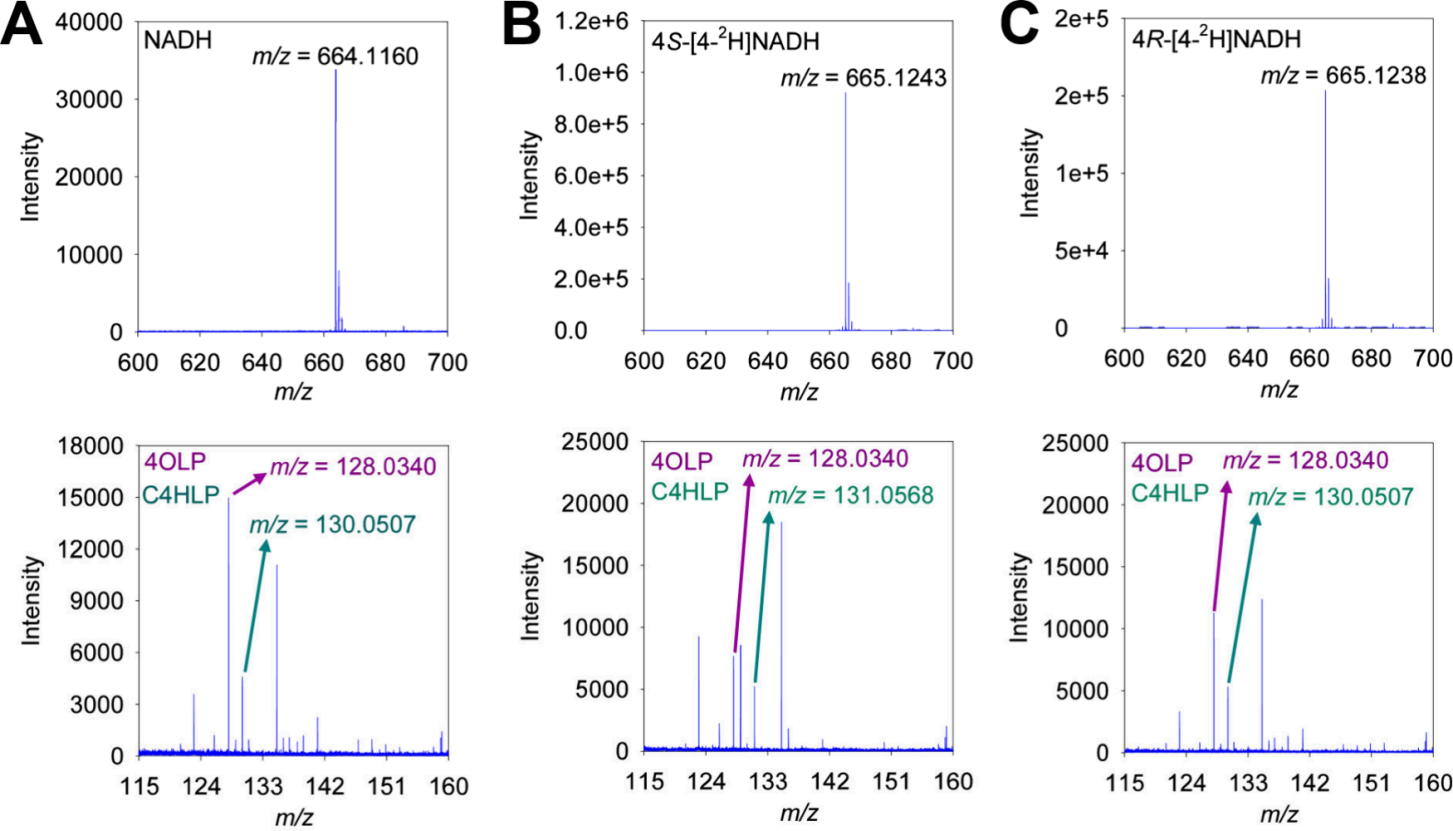
High-resolution ESI-MS spectra in negative mode of the *Hs*BDH2-catalyzed reaction. (A) MS spectra of the reaction of NADH and 4-oxo-l-proline (4OLP) to form *cis*-4-hydroxy-l-proline (C4HLP). (B) MS spectra of the reaction of 4*S*-[4-^2^H]NADH and 4-oxo-l-proline (4OLP) to form *cis*-4-hydroxy-l-proline (C4HLP). (C) MS spectra of the reaction of 4*R*-[4-^2^H]NADH and 4-oxo-l-proline to form *cis*-4-hydroxy-l-proline (C4HLP). In all cases, the analyzed ion was [M–H]^−^.

As expected, when NADH and 4-oxo-l-proline react in the presence of *Hs*BDH2, an *m*/*z* value of 130.0507 corresponding to *cis*-4-hydroxy-l-proline is detected, along with NADH and 4-oxo-l-proline (Figure 6A). Upon reaction of 4*S*-[4-^2^H]NADH and 4-oxo-l-proline in the presence of *Hs*BDH2, an *m*/*z* value of 131.0568 is detected in place of an *m*/*z* value of 130.0507, which would be predicted if a deuteride were transferred, instead of a hydride, to 4-oxo-l-proline (Figure 6B). On the contrary, when 4*R*-[4-^2^H]NADH and 4-oxo-l-proline react in the presence of *Hs*BDH2, an *m*/*z* value of 130.0507 is again detected, in accordance with a hydride, not a deuteride, being transferred in the reaction. These observations are compatible with *Hs*BDH2 catalyzing the stereospecific transfer of the pro-*S* hydrogen from NADH to 4-oxo-l-proline.

### Rate-Limiting Steps in the *Hs*BDH2-Catalyzed Reaction

**Figure 7:**
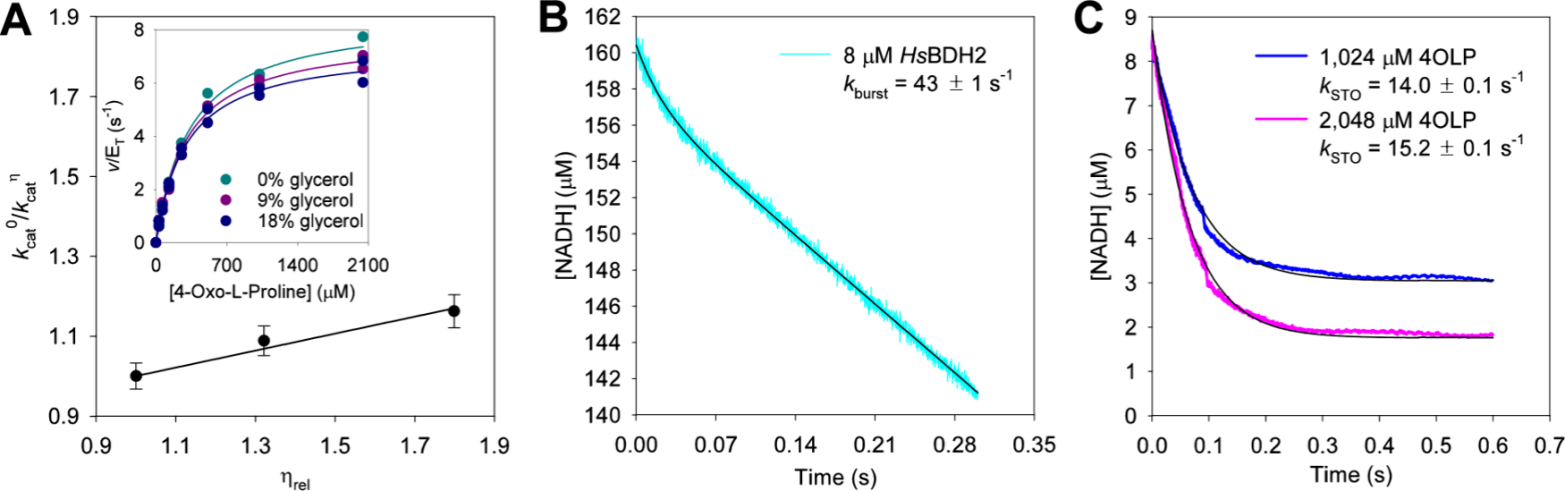
Rate-limiting steps in *Hs*BDH2 catalysis. (A) Solvent viscosity effects on *Hs*BDH2 *k*_cat_. Data points are *k*_cat_ ± fitting error from eq 1. The line is the best fit of the data to eq 8. The inset shows Michaelis–Menten plots in the presence and absence of glycerol, with all data points shown for two independent measurements. Lines are best fits according to eq 1. (B) Approach to steady-state consumption of NADH by *Hs*BDH2. The cyan line shows the data, and the black line is the best fit to eq 9. (C) Single-turnover kinetics of *Hs*BDH2. Blue and pink lines show the data, and black lines are best fits to eq 10.

To start to uncover the contributions of different steps to *k*_cat_, solvent viscosity effects were evaluated by increasing the fraction of microviscogen glycerol in the reaction mixture (Figure 7A). Each *k*_cat_ was obtained from 4-oxo-l-proline saturation curves at different glycerol levels (Figure 7A, inset), and a plot of *k*_cat_ ratios against relative viscosity^22^ (Figure 7A) produced a slope of 0.21 ± 0.02 upon the best fit of the data to eq 8. This points to a modest contribution to the overall rate constant of diffusion of the product from the enzyme.^22^ The macroviscogen polyethylene glycol 8000 (PEG-8000) had no effect on *k*_cat_ (Figure S10A), suggesting glycerol effects are indeed a result of increased solvent microviscosity.^22^ A plot of *k*_cat_/*K*_M_^4OLP^ ratios against relative viscosity had a negligible slope of 0.02 ± 0.01 (Figure S10B), which would indicate no diffusional steps contributing to *k*_cat_/*K*_M_^4OLP^.^22^ However, *k*_cat_/*K*_M_^4OLP^ was moderately affected by PEG-8000 (Figure S10A), pointing to possible effects beyond microviscosity perturbing the second-order rate constant.

To shed light on the contribution of the chemical step to *Hs*BDH2 *k*_cat_, the approach to the steady-state consumption of NADH was evaluated by rapid kinetics under multiple-turnover conditions. The data indicated a pre-steady-state burst of substrate depletion preceding the steady-state phase (Figure 7B). The best fit of the data to eq 9 produced a burst-phase amplitude of 3 μM, a first-order macroscopic rate constant (*k*_burst_) of 43 ± 1 s^–1^ (the sum of all rate constants from the Michaelis complex onward) governing the burst phase, and a first-order macroscopic steady-state rate constant (*k*_SS_) of 6.8 ± 0.1 s^–1^. The measured *k*_SS_ similar to *k*_cat_ and the observation of the preceding burst indicate steps after chemistry make a larger contribution toward limiting the reaction rate.^23^ The reaction was also monitored under single-turnover conditions with 10 μM *Hs*BDH2, 8.3 μM NADH, and two nearly saturating concentrations of 4-oxo-l-proline (Figure 7C). The best fit of the data to eq 10 yielded single-turnover rate constants (*k*_STO_) of 14.0 ± 0.1 and 15.2 ± 0.1 s^–1^ with 1024 and 2048 μM 4-oxo-l-proline, respectively, suggesting the *k*_STO_ reflects a unimolecular rate constant. These *k*_STO_ values indicate that the interconversion between *Hs*BDH2:NADH:4OLP and *Hs*BDH2:NAD^+^:C4HLP ternary complexes is at least twice as fast as *k*_cat_, also pointing to a step after chemistry contributing the most to the overall reaction rate.

## Discussion

The work redefining the activity of *Hs*BDH2 as a 4-oxo-l-proline reductase that generates *cis*-4-hydroxy-l-proline^7^ has opened the door to the exploration of the biochemistry of this enzyme and its catalyzed reaction in more detail, motivated not only by the elusive biochemical fate of *cis*-4-hydroxy-l-proline^7^ but also by the anticancer activity of *cis*-4-hydroxy-l-proline.^9,10^ The characterization of the kinetics and thermodynamics of the reaction presented here provides the first in-depth analysis of the *Hs*BDH2-catalyzed reduction of 4-oxo-l-proline, a crucial step toward elucidating the catalytic mechanism of *Hs*BDH2 and helping inform the future development of specific chemical tools to explore the function of this enzyme in healthy and cancer cells. For instance, the tight binding of NADH poses challenges for compounds aiming to compete with NADH for the enzyme but instead provides an avenue for uncompetitive inhibitors with regard to NADH, which would bind to the *Hs*BDH2:NADH binary complex.

**Scheme 2:**
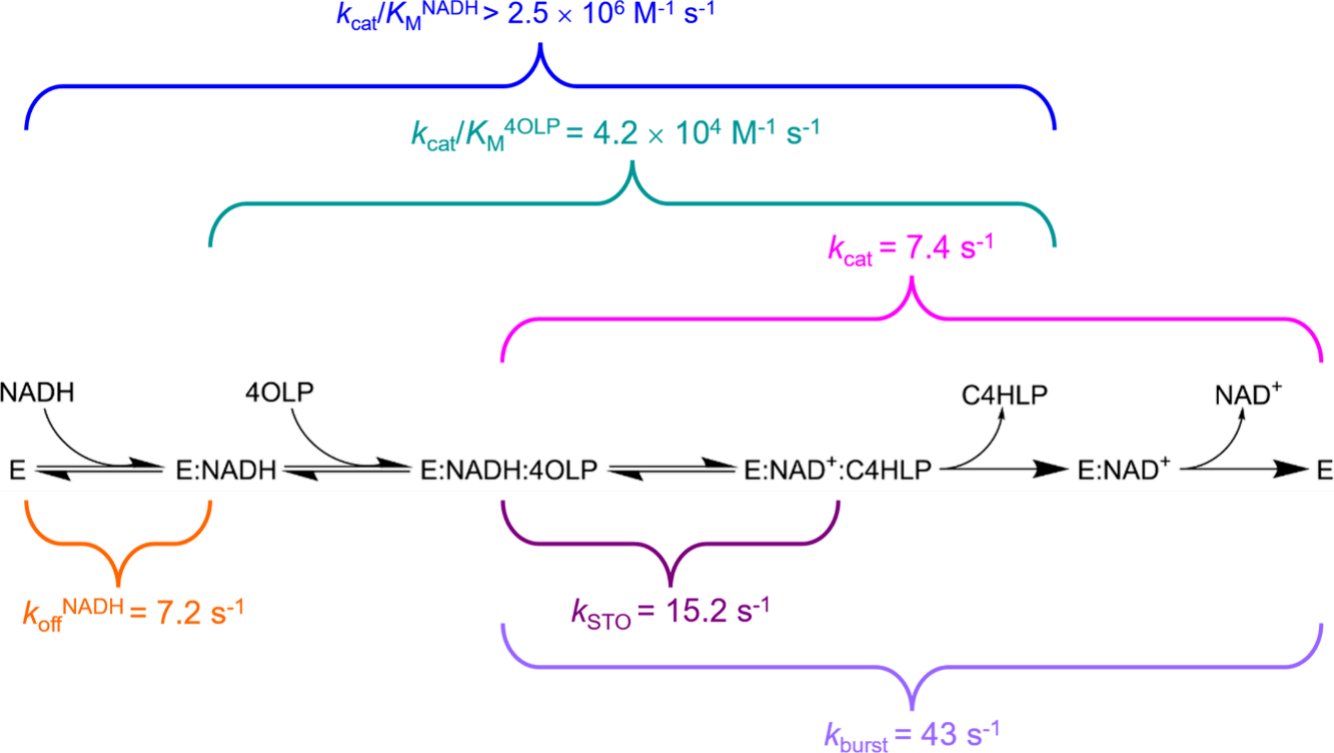
Proposed Kinetic Sequence for the *Hs*BDH2 Reaction and Associated Rate Constants

Scheme 2 summarizes the proposed kinetic sequence for *Hs*BDH2 based on findings reported in this work, along with the rate constants measured here and the reaction steps they encompass under conditions under which accumulation of the free product is negligible. While the kinetic mechanism could in theory still be random, the ordered mechanism with NADH as the first substrate to bind and NAD^+^ as the last product to dissociate is likely operational in practice. This is supported by the following observations: tight binding between the enzyme and NADH that leads to purification of the *Hs*BDH2:NADH complex, the ability of NAD^+^ to bind to the apoenzyme, and the very high *K*_M_ for *cis*-4-hydroxy-l-proline in the reverse reaction direction.

The group with an apparent p*K*_a_ of 7.3 that must be deprotonated for the optimum binding of 4-oxo-l-proline to *Hs*BDH2 and/or catalysis may be either an enzyme residue or the substrate itself, for instance, the α-amino group of 4-oxo-l-proline. The putative p*K*_a_ of 8.7 for a group that must be protonated for maximum binding and/or catalysis falls in the range of the catalytic triad Tyr residue in SDRs,^24^ which often act as general acids to donate a proton to the substrate during the reduction reaction.^6,24^ Future site-directed mutagenesis studies on *Hs*BDH2 may further inform the role of acid–base catalysis in the reaction.

The establishment of the stereochemistry of hydride donation as the pro-*S* hydrogen being transferred from NADH matches that of related (*R*)-3-hydroxybutyrate dehydrogenases^6^ and β-ketoacyl-ACP reductases,^24,25^ SDR enzymes that also catalyze NAD(P)H-dependent reductions of carbonyl groups. This is also compatible with the crystal structure of NAD^+^-bound *Hs*BDH2, as the orientation of the nicotinamide moiety would position it opposite the docked substrate (*R*)-3-hydroxybutyrate in an arrangement conducive to transfer of the pro-*S* hydrogen between the two molecules.^1^

*Hs*BDH2 binds tightly to NADH at 25 °C (*K*_D_ = 0.48 μM), reminiscent of other NAD(P)H-dependent reductases, the most studied of which is dihydrofolate reductase (DHFR), with a *K*_D_ of 0.33 μM reported for *E. coli* DHFR.^26^ At 5 °C, the *k*_off_ of 0.13 s^–1^ leads to co-purification of NADH with *Hs*BDH2, while at 25 °C, it increases to 7.2 s^–1^, in range of the *k*_off_ for NADPH found for *E. coli* DHFR (3.5 s^–1^)^26^ and for *Mycobacterium tuberculosis* DHFR (1.7 s^–1^), although in the latter example NADPH dissociates from the ternary complex with one of the products.^27^ It should be pointed out that the competition experiments carried out here yield an overall *k*_off_ for NADH and do not inform the number of steps in the dissociation mechanism.^17^

Intriguingly, the *Hs*BDH2 *T*_m_ of ∼44 °C in the absence of added NADH is close to the physiological temperature of 37 °C, and some denaturation could be expected at this temperature. This problem is likely solved by the thermostability conferred upon binding to NADH. The *T*_m_ of ∼33 °C of the *Hs*BDH2 apoenzyme falls below the physiological temperature, but it is stabilized to ∼47 °C in the presence of 1.2 mM NAD^+^. The combined concentration of NADH and NAD^+^ in a mammalian cell is estimated to vary between 0.3 and 1.0 mM,^28^ and NADH/NAD^+^ ratios between 1/10 and 1/700,^29,30^ as reported by Walsh *et al*.^31^ In the cytoplasm, where *Hs*BDH2 is localized in the cell,^1,2,7^ the estimated ratio is between 1/700 and 1/800, as most of the NADH is found in mitochondria.^32^ Assuming that this extends to human cells, the lowest expected NADH levels would yield ∼0.38 μM NADH (just below the *Hs*BDH2 *K*_D_ at 25 °C) and ∼300 μM NAD^+^ (∼10-fold greater than the *Hs*BDH2 *K*_D_ at 25 °C), and the highest expected levels would lead to 1.45 μM NADH (∼3-fold greater than the *Hs*BDH2 *K*_D_ at 25 °C) and ∼1 mM NAD^+^ (∼34-fold greater than the *Hs*BDH2 *K*_D_ at 25 °C). Therefore, *Hs*BDH2 may have evolved to be always bound to either NAD^+^ or, to a lesser extent, NADH in the cell, which might explain the significant increase in thermostability resulting from coenzyme binding. High thermostability upon NADH binding is also a feature of a cold-adapted (*R*)-3-hydroxybutyrate dehydrogenase.^33^ In *E. coli*, the estimated NADH concentration is ∼83 μM,^34^ enough to saturate recombinant *Hs*BDH2.

Pre-steady-state kinetic analysis under multiple- and single-turnover conditions indicates that a slow step after hydride transfer contributes to the *Hs*BDH2 *k*_cat_. This step may be associated with product release, and a modest contribution to the reaction rate from diffusion of one of the products from the enzyme is inferred from solvent viscosity effects. This scenario was also found with cold- and warm-adapted orthologues of (*R*)-3-hydroxybutyrate dehydrogenase catalyzing the reduction of their physiological substrate acetoacetate.^33^ It should be pointed out that even the *k*_STO_ measured for *Hs*BDH2 reflects the rate of interconversion between ternary complexes with substrates and products and might not reflect the rate of chemistry itself. For instance, with (*R*)-3-hydroxybutyrate dehydrogenases, even *k*_STO_ may have contributions from physical steps (e.g., conformational changes), as revealed by pre-steady-state kinetic isotope effects.^35^ Future isotope effect studies will be useful in establishing the mechanism of hydride transfer by *Hs*BDH2 and its contribution to the overall reaction rate.

## Conclusions

In summary, *Hs*BDH2 binds tightly to NADH, which imparts temperature stability to the enzyme, and transfers the C4 pro-*S* hydrogen from the coenzyme to 4-oxo-l-proline to generate *cis*-4-hydroxy-l-proline, a compound with known anticancer properties. The interconversion of the ternary complexes of the enzyme and substrates and the enzyme and products is faster than at least one subsequent step, which has a modest contribution from product diffusion. To unveil details of the chemical step and its contribution to the limiting *k*_cat_, kinetic deuterium isotope effect studies may prove to be valuable.

## Supporting Information Available

Further experimental results on *Hs*BDH2 biochemistry, including SDS–PAGE, mass spectrometry, UV–VIS spectroscopy, and kinetic analysis (PDF)

## Accession Codes

*Hs*BDH2, UniProt Q9BUT1.

## Abbreviations

BDH2: 4-oxo-l-proline reductase
*Hs*BDH2: human BDH2
ITC: isothermal titration calorimetry
DSF: differential scanning fluorimetry
LC-ESI-MS: liquid chromatography-electrospray ionization-mass spectrometry
*k*_off_: dissociation rate constant
*T*_m_: melting temperature
*K*_D_: equilibrium dissociation constant
Δ*G*: Gibbs free energy
*K*_eq_: equilibrium constant
PEG-8000: polyethylene glycol 8000
*k*_STO_: single-turnover rate constant
*k*_burst_: burst-phase rate constant
*k*_SS_: steady-state rate constant
DTT: dithiothreitol
IPTG: isopropyl β-d-1-thiogalactopyranoside
MES: 2-(*N*-morpholino)ethanesulfonic acid
CHES: 2-(cyclohexylamino)ethanesulfonic acid
HEPES: 4-(2-hydroxyethyl)piperazine-1-ethanesulfonic acid
DHFR: dihydrofolate reductase
