# Supplementary material for: Kinetic and Thermodynamic Characterization of Human 4-Oxo-l-proline Reductase Catalysis
Source: Biochemistry. 2025 Jan 30;64(4):860–70. doi: 10.1021/acs.biochem.4c00721 (PMC11840923; doi:10.1021/acs.biochem.4c00721)
Supplement: Supplementary file 1 — bi4c00721_si_001.pdf [file bi4c00721_si_001.pdf]

Supporting Information

Kinetic and thermodynamic characterisation of human 4-oxo-L-proline reductase catalysis

Ennio Pečaver[†], Greice M. Zickuhr[§], Teresa F. G. Machado[¥], David J. Harrison[§,£], Rafael G. da Silva[†,*]

[†]School of Biology, Biomedical Sciences Research Complex, University of St Andrews, St Andrews, KY16 9ST, United Kingdom

[§]School of Medicine, University of St Andrews, St Andrews, KY16 9TF, United Kingdom

[¥]EaStCHEM School of Chemistry, Biomedical Sciences Research Complex, University of St Andrews, St Andrews, KY16 9ST, United Kingdom

[£]NuCana Plc, Edinburgh EH12 9DT, United Kingdom

*To whom correspondence may be addressed: rgds@st-andrews.ac.uk, phone: +44 01334 463496

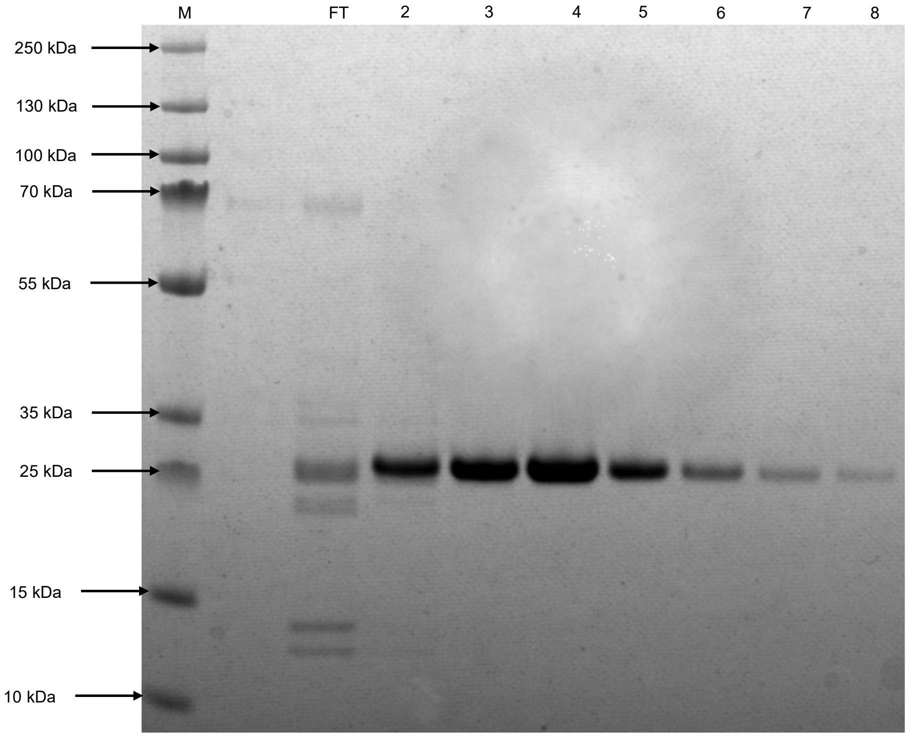

Figure S1. SDS-PAGE analysis of NADH-bound *Hs*BDH2 fractions (lanes 2 – 8) eluted from the HisTrap FF column. FT is the flow through, and M is the PageRuler Plus Prestained molecular weight marker.

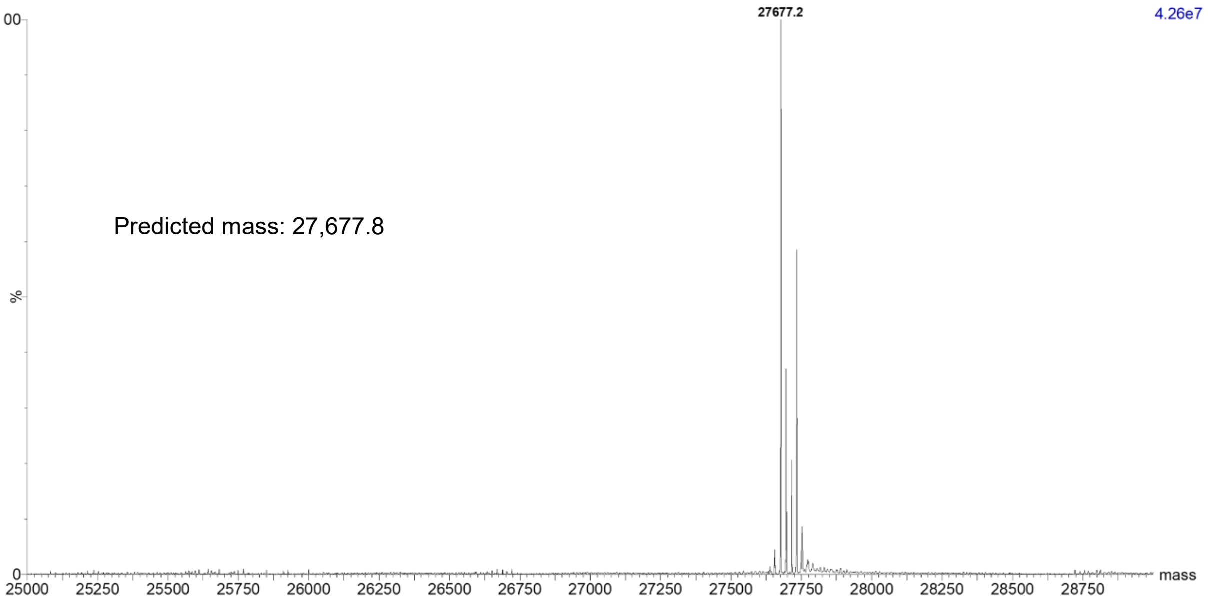

Figure S2. LC-ESI-MS analysis of purified *Hs*BDH2.

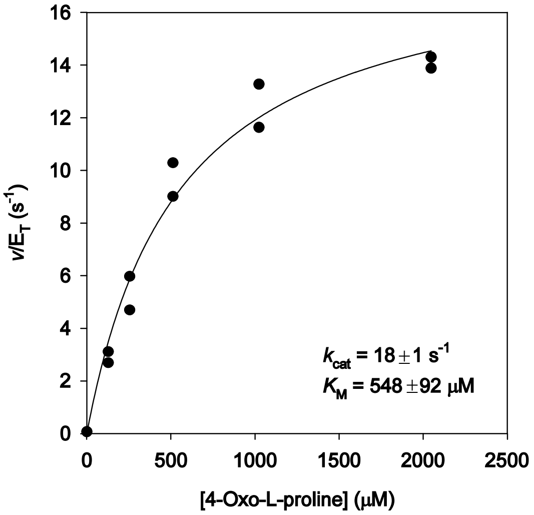

Figure S3. Michaelis-Menten plot for 4-oxo-L-proline under experimental conditions (e.g. 37 °C, phosphate buffer pH 6.5) matching those previously reported [7]. All data points are shown for two independent measurements. Line is best fit of the data to equation 1.

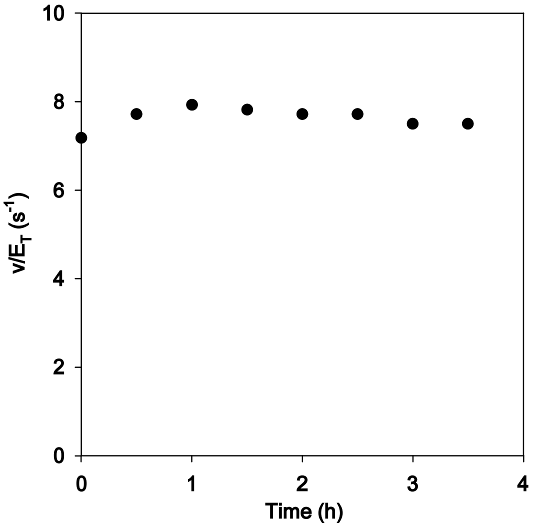

Figure S4. *Hs*BDH2 catalytic activity at 25 °C upon incubation of the enzyme at 25 °C for 3.5 h. Data points are the mean of five measurements.

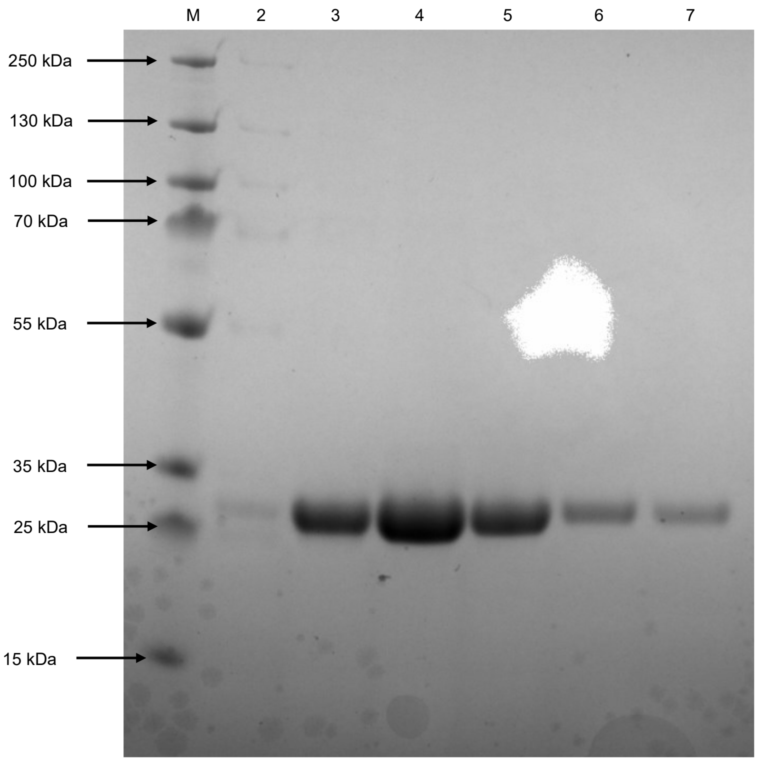

Figure S5. SDS-PAGE analysis of apo-*Hs*BDH2 fractions (lanes 3 – 7) eluted from the HisTrap FF column. Lane is the flow through, and M is the PageRuler Plus Prestained molecular weight marker.

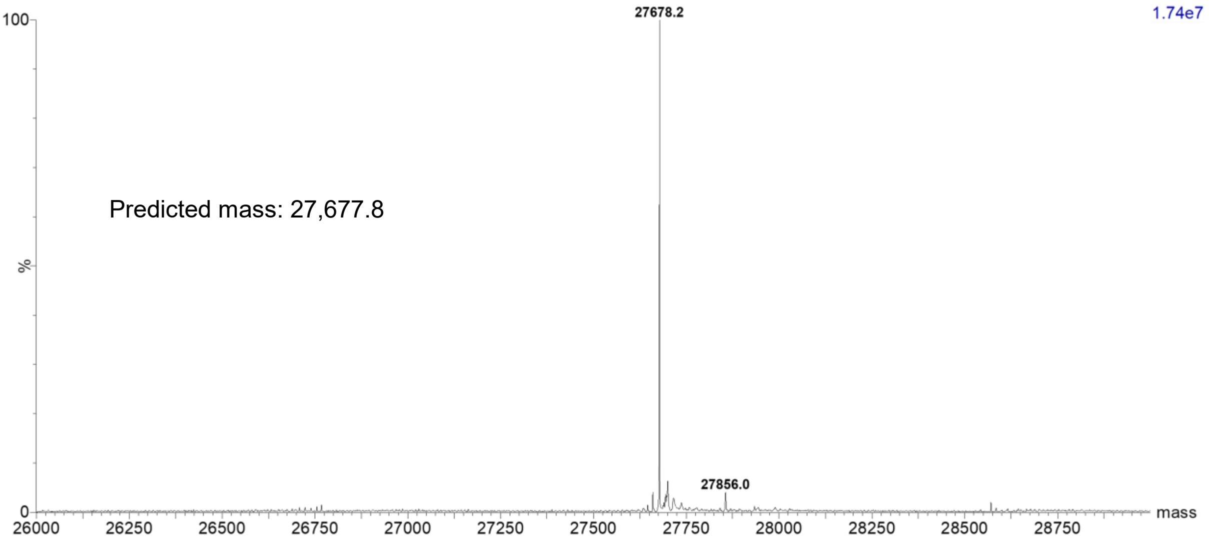

Figure S6. LC-ESI-MS analysis of purified apo-*Hs*BDH2.

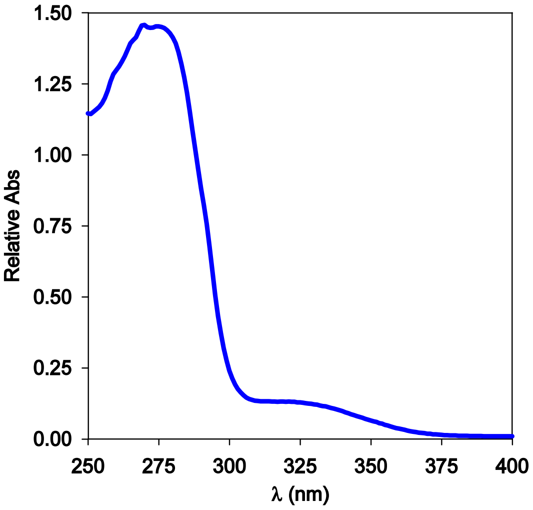

Figure S7. UV-VIS spectrum of purified *Hs*BDH2 "apoenzyme".

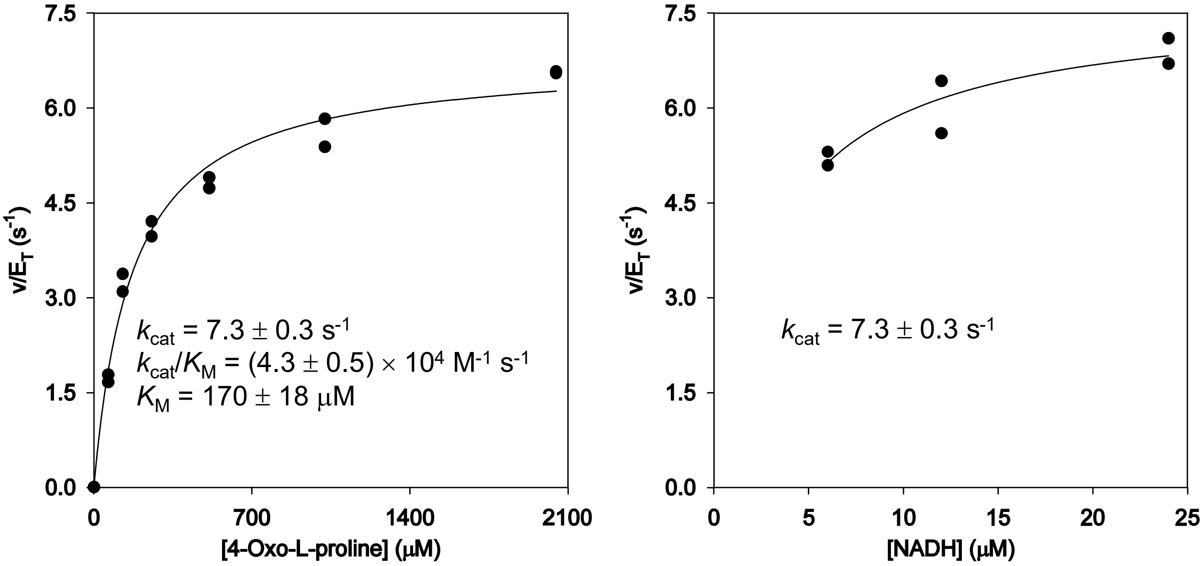

Figure S8. Michaelis-Menten plots for 4-oxo-L-proline and NADH with *Hs*BDH2 apoenzyme. All data points are shown for two independent measurements. Line is best fit of the data to equation 1.

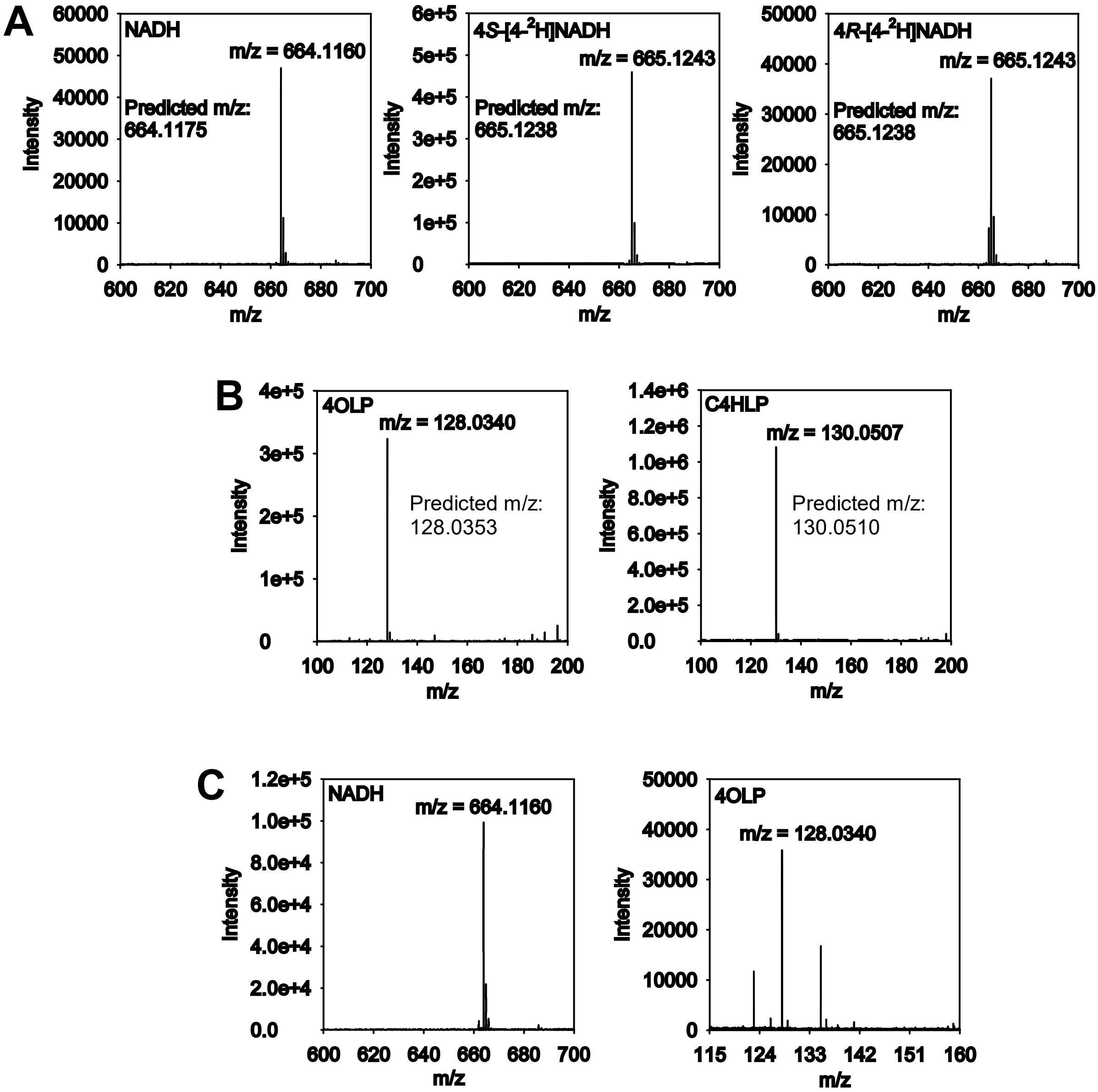

Figure S9. High-resolution ESI-MS spectra in negative mode of standards and reaction control. **(A)** MS spectra of commercial NADH and synthesised 4*S*-[4-^{2}H]NADH and 4*R*-[4-^{2}H]NADH. **(B)** MS spectra of commercial 4-oxo-L-proline (4OLP) and *cis*-4-hydroxy-L-proline (C4HLP). **(C)** MS spectra of the reaction of NADH and 4-oxo-L-proline in the absence of *Hs*BDH2. In all cases, the analysed ion was [M–H]$^-$.

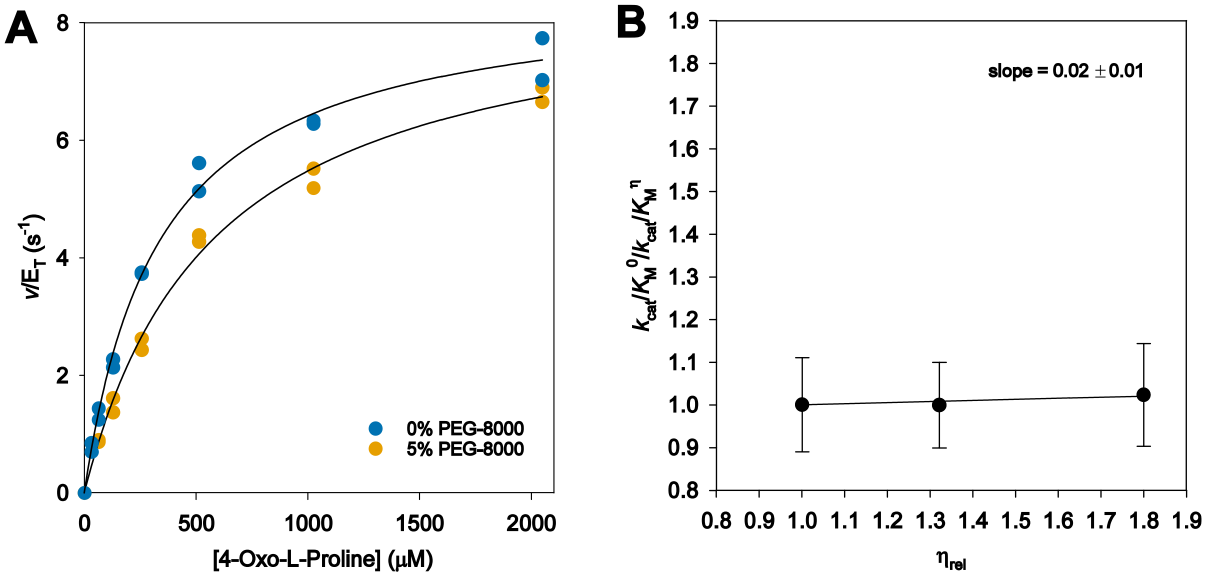

Figure S10. Solvent viscosity effects. **(A)** Michaelis-Menten plots for 4-oxo-L-proline in the presence and absence of PEG-8000. All data points are shown for two independent measurements. Line is best fit of the data to equation 1, yielding k_{cat} of 8.6 ± 0.2 s^{-1} and 8.6 ± 0.3 s^{-1} at 0% and 5% PEG-8000, respectively, and k_{cat}/K_M^{4OLP} of 25,519 ± 2,023 M^{-1} s^{-1} and 14,853 ± 1,265 M^{-1} s^{-1} at 0% and 5% PEG-8000, respectively. **(B)** Solvent viscosity effect on k_{cat}/K_M^{4OLP}. Data are fitted value ± fitting error from equation 1 fitted to the data in Figure 7A, inset. Line is best fit of the data to equation 9.
